# An algorithm for generating biophysically realistic three‐dimensional arteriolar networks applied to rat skeletal muscle

**DOI:** 10.14814/phy2.70704

**Published:** 2025-12-19

**Authors:** Yuki Bao, Jefferson C. Frisbee, Daniel Goldman

**Affiliations:** ^1^ Department of Medical Biophysics University of Western Ontario London Ontario Canada; ^2^ Department of Physiological Sciences, College of Veterinary Medicine Oklahoma State University Stillwater Oklahoma USA

**Keywords:** arteriolar network, biosimulation, computational modeling, fractal, geometry, microcirculation, perfusion distribution, topology

## Abstract

The microcirculation comprises small vessel networks that regulate blood perfusion within tissues. The relationship between tissue shape or size and its microvascular properties is not yet clear. This study develops an algorithm for computationally simulating branching arteriolar networks within ellipsoidal tissue volumes, including user‐adjustable parameters (e.g., tissue width‐length‐height dimensions and microvessel density) for application within different rodent skeletal muscles. The algorithm is developed using principles from constrained constructive optimization, an iterative network generation framework based on proposed mechanisms of vascular growth. Networks generated within muscles of varying shapes and sizes were analyzed over a range of geometric (e.g., mean diameter, length, and number of bifurcations per Strahler's and centrifugal order, fractal dimension) and hemodynamic (e.g., Murray's law exponent, hematocrit) properties. Statistical similarity was observed across different skeletal muscle tissues, with differences due to tissue shape being observed only above a vessel diameter threshold of ~25 μm (varying at large or small tissue volumes at the scale m^3^ or mm^3^). The algorithm was comprehensively validated against in vivo data using different modeling approaches (whole tissue vs. subsection simulations). The algorithm's accuracy and adaptability support a wide range of research objectives and contributes to advancing current understanding of perfusion distribution in healthy tissue.

## 1 INTRODUCTION

The microcirculation consists of small blood vessels (<100–200 μm in diameter) that are typically found embedded in tissues. These vessels are commonly grouped by structural or functional differences (e.g., arterioles, capillaries, and venules) that integrate synergistically to ensure adequate tissue perfusion. There is abundant evidence that arterioles, which are recognized by a higher relative composition of smooth muscle in their walls, are predominantly responsible for blood flow regulation in tissues and creating the observed perfusion distribution patterns (Mironova et al., 2024). As such, these vessels are central to discussions connecting observed tissue perfusion with health outcomes. Tissues come in a wide variety of shapes and sizes and require distinct vascular structures to yield perfusion patterns that accommodate their unique metabolic needs. For example, skeletal muscles have extensive arteriolar networks to accommodate higher metabolic states like exercise; meanwhile, the cornea's limited vascularization is essential for the cornea's transparency and unobstructed vision (Augustin & Koh, 2017). Understanding the relationship between tissue geometry and its vasculature is essential, particularly in fields such as tissue engineering, where arteriolar networks must support the metabolic demands of both the engineered tissue and host prior to implantation (Yang et al., 2020). It is also important in disease research and therapy development, as arteriolar control of perfusion is highly responsive to local physiological changes and can often be impaired in disease (Feuer et al., 2022; Frisbee et al., 2011). Impaired arteriolar control can contribute to disease progression by constraining tissue function or inducing damage due to inadequate perfusion (e.g., hypoxia). This has been observed across numerous conditions, including ischemic heart disease, renal failure, stroke, diabetes, pulmonary hypertension, and dementia (Feuer et al., 2022; Thompson & Hakim, 2009).

Past research on microvascular properties in tissues has been primarily grounded in experimental observation. These observations have led to major theoretical concepts, such as Murray's law (Murray, 1926) and the Fahraeus effect (Fåhraeus, 1929; Pries et al., 1990), which have supported quantitative understanding of microvascular geometry and hemodynamics. While experimental work remains critical, it is commonly constrained by factors such as being labor‐intensive, technically challenging, invasive, and costly. As such, there remains a lack of quantitative data on microvascular network properties and their relationship to tissue geometry and function in the literature. Existing studies typically cover a narrow range of vessel diameters (~10–30 μm) or focus on select vascular metrics. Given these limitations, computational modeling has become an increasingly valuable approach for studying microvascular function and tissue perfusion. These models are typically built based on experimental findings and have been applied to a range of topics, such as red and white blood cell dynamics or cellular and vessel responses to various stimuli (C. Arciero et al., 2017; Fenton et al., 1985; Secomb, 2008). Despite their broad potential, existing models are often computationally demanding or require significant user expertise, limiting accessibility for investigators not deeply invested in the approach. Furthermore, many models operate within a defined, static vascular geometry, making them study‐specific and poorly applicable to other tissues. A mathematical model of microvascular network geometry, topology, and hemodynamics that is adaptable for different tissues and accessible to investigators would be a greatly beneficial tool for exploring fundamental questions as well as diverse applications to disease and other challenges.

In recent work, we developed a computational algorithm for generating branching arteriolar networks within a two‐dimensional (2D) circular tissue domain (Bao et al., 2024). The algorithm featured user‐adjustable parameters, enabling customization of network properties to reflect tissue‐specific microvascular characteristics (e.g., Murray's law exponent (Kassab, 2006)). Validation efforts comparing arteriolar networks generated in rat skeletal muscle to experimental data demonstrated that the algorithm produced visually and statistically realistic results. As a result, the algorithm was able to yield valuable insight into tissue perfusion distributions caused by network geometry and hemodynamics. This is a fundamental step before further considerations of regulation of vascular tone due to varying local stimuli and network‐wide dynamic remodeling. Though the previously described 2D circular tissue domain was a reasonable approximation for certain vascular territories, extending the developed algorithm to enable vascularization of a customizable three‐dimensional (3D) space would significantly broaden applicability to different tissues. This advancement would allow more anatomically accurate modeling and facilitate explorations on the relationship between tissue geometry and its microvascular properties.

As such, the present study builds off previous work (Bao et al., 2024) to present an extended computational algorithm for generating realistic branching arteriolar networks in 3D ellipsoidal skeletal muscle tissue volumes. As in previous work (Bao et al., 2024), the algorithm is based on the principles of constrained constructive optimization (CCO), a step‐by‐step approach of network construction that fills in tissue space comparable to proposed mechanisms of real‐life vascular growth (Schreiner & Buxbaum, 1993). This algorithm features user‐adjustable parameters that allow generated vasculature to be tailored to different tissue‐specific vascular (e.g., microvessel density) and geometric (e.g., tissue width‐length‐height dimensions) properties. A key focus of this study is exploring the relationship between tissue geometry and its microvascular properties using the developed algorithm: arteriolar networks will be sampled from vasculature generated in tissues of varying shapes and sizes and comprehensively analyzed over a range of geometric and hemodynamic properties for intra‐ and inter‐tissue differences. These differences will provide insight into how arteriolar hemodynamics and geometry influence tissue perfusion distributions when under different tissue constraints, which will be valuable for various areas in vascular research and is otherwise difficult to explore experimentally. Finally, the algorithm will be validated by comparing arteriolar networks generated in tissues characteristic of rat skeletal muscle to experimental data. If the generated networks resemble in vivo networks across a range of geometric and hemodynamic properties, the algorithm will be considered sufficiently accurate for practical usage. Validation will include measurements of the rat gluteus maximus arteriolar networks from previous work (Al Tarhuni et al., 2016; Al‐Khazraji et al., 2012), which remains one of the most detailed datasets available for microvascular geometry and topology in the literature.

## 2 MATERIALS AND METHODS

### 2.1 Network generation algorithm

This algorithm generates branching arteriolar tree networks in 3D ellipsoidal tissue volumes with user‐adjustable width‐length‐height dimensions to capture most common tissue shapes. Vessels within the generated networks were approximated as a sequence of straight cylindrical tubes connected in series; this is a commonly implemented approximation for simplifying the calculation of hemodynamic parameters (e.g., blood flow and pressure) along the length of a vessel. Previous studies have recognized the assumption as accurate on average when comparing calculated hemodynamics to experimental data (Pries et al., 1990, 1997), which is conducive for goals of producing networks that are broadly representative of the microcirculation of tissues. Additional user‐adjustable parameters were implemented in the algorithm so that generated vasculature may adopt microvascular properties belonging to different tissues of interest. Default values for certain user‐adjustable parameters were specified based on experimental microvascular data from the rat gluteus maximus muscle (Al Tarhuni et al., 2016; Al‐Khazraji et al., 2012); rationale for these default values is later described in the Algorithm Validation section. A descriptive list of the user‐adjustable parameters implemented in the algorithm is provided in Table 1.

**TABLE 1:** A list of user‐adjustable parameters used in the algorithm, with their definitions and default values.

Variable names	Default value	Definition
*Nbif*	N/A	The number of bifurcations in the generated network, i.e., network density.
*Qperf*	0.12 nL/s	Blood flow within each terminal arteriole.
*dPtot*	30 mmHg	The pressure gradient across the network from inlet to terminal arteriole.
*Vperf*	1.8 mm^3^	Tissue volume perfused per terminal arteriole, i.e., the tissue's vessel density.
*Rx*, *Ry*, *Rz*	N/A	The ratio of the width, length, and height of the tissue, respectively. Used to specify the ellipsoidal tissue's dimensions.
γ	2.63	The Murray's law exponent, which characterizes the power law relationship between diameter and blood flow.
*expVal*	0 (off)	A percentage value, where any daughter vessel with a diameter which is less than this percentage of the parent's diameter is ignored when calculating network properties.

The algorithm uses principles of CCO to generate vasculature. As such, vessels are added to the network in a step‐by‐step fashion and the tissue grows to accommodate every newly added vessel, until the final network and tissue size is reached. At every iteration, vessels are added to the network at an “ideal” location, which consists of optimizing for both the ideal vessel in the existing network for the newly added vessel to connect to and the ideal bifurcation angles for the newly formed bifurcation to possess. This optimization will be based on attempting to minimize overall blood volume in the network, assuming blood is an expensive resource that is conserved in real‐life vascular growth (Schreiner et al., 1995). Concisely, the algorithm may be described as follows:

1. Specify the desired tissue geometry and network properties using the user‐adjustable parameters. Most user‐adjustable parameters have default values offering a decent approximation for most skeletal muscle tissues. Parameters without default values that will require attention are *Nbif* (the number of bifurcations, i.e., the network's density/size) and *Rx*, *Ry*, *Rz* (the tissue's dimensions).
2. Set an initial arteriole from which the rest of the network will grow.
  - Specify the starting location within the tissue geometry from which the network will start growing. This starting location may also be understood as where the inlet arteriole enters the tissue.
  - Connect the starting location to a randomly selected point within the tissue volume. This point may be understood as a location within the tissue that requires blood perfusion. The initial arteriole's other properties (e.g., diameter, blood flow, etc.) will be calculated by the algorithm based on the previously set user‐adjustable parameters
3. Repeat the below steps 3A to 3D until the vascular network has sufficiently filled in the tissue geometry (i.e., the number of bifurcations in the network sum to *Nbif*):
  C If the number of bifurcations in the network has reached *Nbif*, stop the algorithm. Otherwise, in preparation to continue growing the network, increase the tissue volume by *Vperf* to accommodate the perfusion volume of one more terminal arteriole.
  D Randomly select a point within the tissue volume that is relatively distant from the nearest vessel. This point may be understood as a location within the tissue that hypothetically requires blood perfusion.
  E Create a new vessel which connects the selected point in step 3B to an “ideal” existing vessel in the network; the “ideal” vessel is identified based on whichever existing vessel yields a minimum total network blood volume when a new vessel connects to it from the selected point.
  F Optimize the angles of the bifurcation formed by the new vessel and the “ideal” existing vessel to further minimize total network blood volume.

**FIGURE 1:**
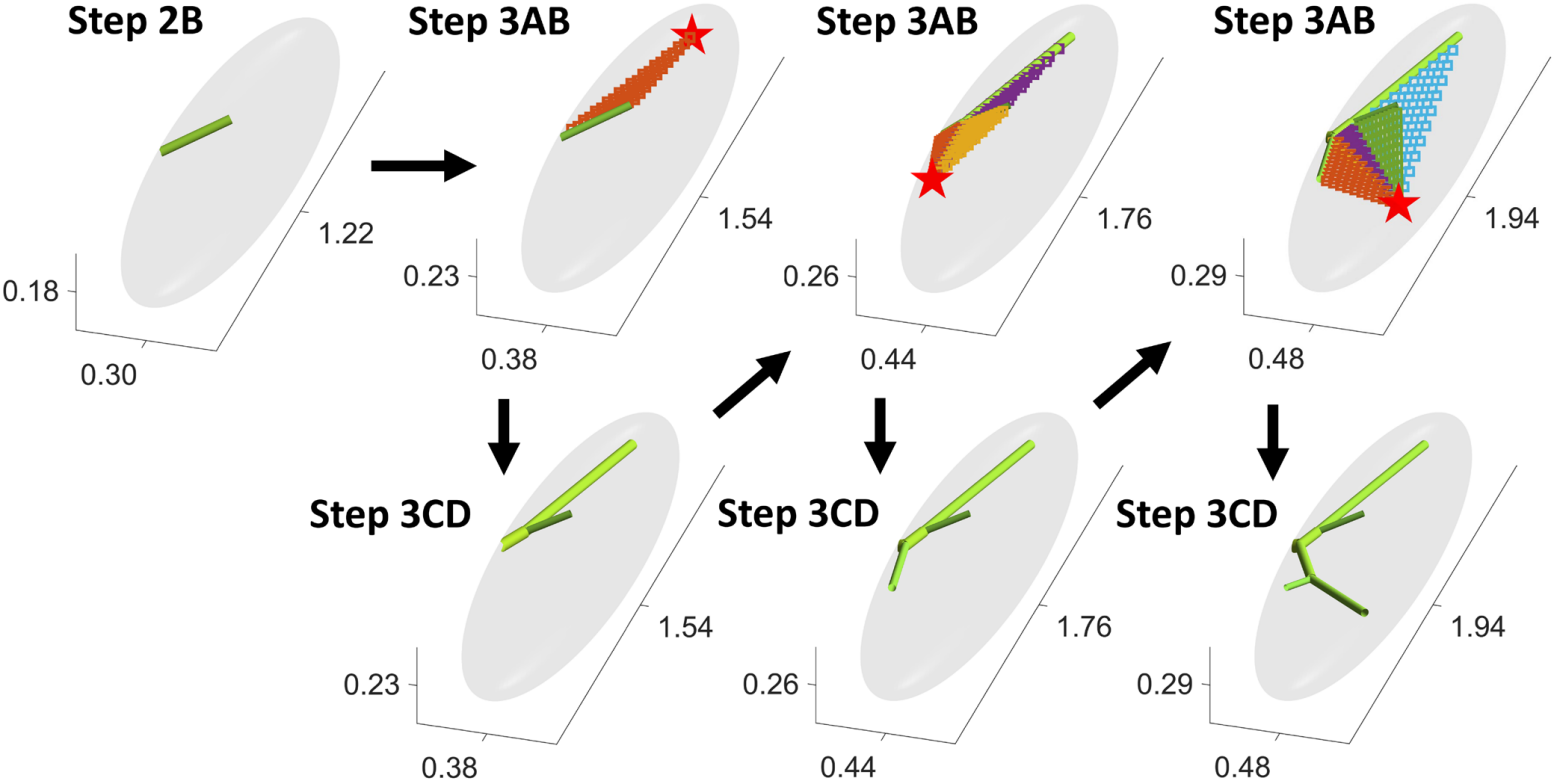
The algorithm presented in a step‐by‐step fashion, starting from the completion of setting the initial vessel (Step 2B). The gray ellipsoid depicts the tissue volume within which network growth is constrained. The axes are labeled based on the width, length, and height of the tissue volume for some arbitrary unit. Vessels are represented as green cylinders. In Step 3B, a point of perfusion (red star) is randomly sampled within the tissue volume; it may be noticed from the changing axes values that the tissue volume width, length and height also scale up concurrently with each sample point of perfusion (Step 3A). The squares represent bifurcation points, each colored for connecting the sampled point (red star) to a different existing vessel in the network. The square that yields a minimum total network blood volume will be identified as the optimal bifurcation point, which will simultaneously determine which existing vessel to connect the sampled point to (Step 3C), and the appropriate bifurcation angles (Step 3D). Step 3A–D will repeat until the user‐specified number of bifurcations (*Nbif*) is reached in the network; Figure 1 depicts three iterations of the algorithm (*Nbif* = 3).

For further clarity, Figure 1 illustrates the algorithm in a step‐by‐step fashion. The random selection of perfusion points (steps 2B and 3B) allows for the generation of networks that are different with every run of the algorithm but statistically similar, provided no other parameters are changed. This randomness was implemented to account for interindividual variations of the same vascular territory. Additionally, the random selection of points was restricted from being outside a certain distance of existing vessels in the network. This is to ensure the network grows in a fashion that fills in the tissue space efficiently, assuming the tissue has a uniform need for perfusion across its volume. The distance was set to $d_{\text{thresh}}=\sqrt{\pi r^{2}/n_{\text{term}}}$ based on suggestions from Schreiner and Buxbaum (Schreiner & Buxbaum, 1993), where *r* is the radius of a spherical tissue of the same volume and *n*_*term*_ is the number of terminal vessels within the generated network. Despite the distance constraint, the resulting distribution of perfusion points is uniform across the tissue. It may also be noted that, although the tissue volume increases by with every iteration of network growth (step 3A), the relative rate of growth (1/*Nbif*) will approach zero as the number of bifurcations becomes large.

When optimizing the generated network, total network blood volume was calculated for every network configuration formed when connecting a new vessel to every existing vessel in the network (step 3C), and for all possible bifurcation angles formed between the new and existing vessels within a limited solution space (step 3D). The network configuration which yields the minimum total network blood volume was chosen at every step of network growth. Total network blood volume may be calculated assuming vessels are consecutively joined straight cylindrical tubes, given lengths, as determined by the chosen network configuration, and diameters, as calculated from a two‐step process where diameters are converged upon such that Murray's law is obeyed and all terminal vessels have a blood flow of *Qperf* (Table 1) within a 1% error tolerance. This two‐step process has been described in detail in previous work (Bao et al., 2024) and yields diameters and blood flow for all vessels in the generated networks, assuming a relative blood viscosity of 3 (Pries et al., 1992). Additionally, blood viscosity and hematocrit were calculated for all vessels using previously described flow simulation methods (Bao et al., 2024), assuming an inlet hematocrit of 0.4 (Pries et al., 1990).

In the optimization process, several additions were made to significantly improve computation time. Firstly, potential network configurations with vessel overlap were labeled as invalid, as these solutions are biologically expensive (high total network blood volume). Secondly, when searching for the optimal existing vessel to connect to (step 3C), total network blood volume was only calculated and compared for the 12 closest vessels to the new point of perfusion. This was implemented under the assumption that it is more biologically efficient (lower total network blood volume) for proximal vessels to perfuse locations in the tissue that require blood. Parallel computing was also implemented in this step using the Parallel Computing Toolbox in MATLAB (MathWorks, n.d.), such that the statistics of each of 12 vessels were calculated in tandem using all available processing units in the CPU. Thirdly, when optimizing the angles of the new bifurcation (step 3D), the triangular space formed by the old existing vessel and the new perfusion point was discretized into multiple possible solutions for the point of bifurcation. This triangular space was defined under the assumption that the point of bifurcation that would yield an ideal bifurcation angle (lowest total network blood volume) would lie within. The triangular space was discretized to 66 points, or 66 possible solutions of bifurcation angles; higher resolution discretization (more than 66 points) did not seem to significantly impact the generated results. The lower resolution discretization of the triangular space saves a significant amount of computation time compared to finding the ideal bifurcation angle solution across the entire tissue volume at a higher resolution.

### 2.2 Calculation of geometric and hemodynamic statistics

**FIGURE 2:**
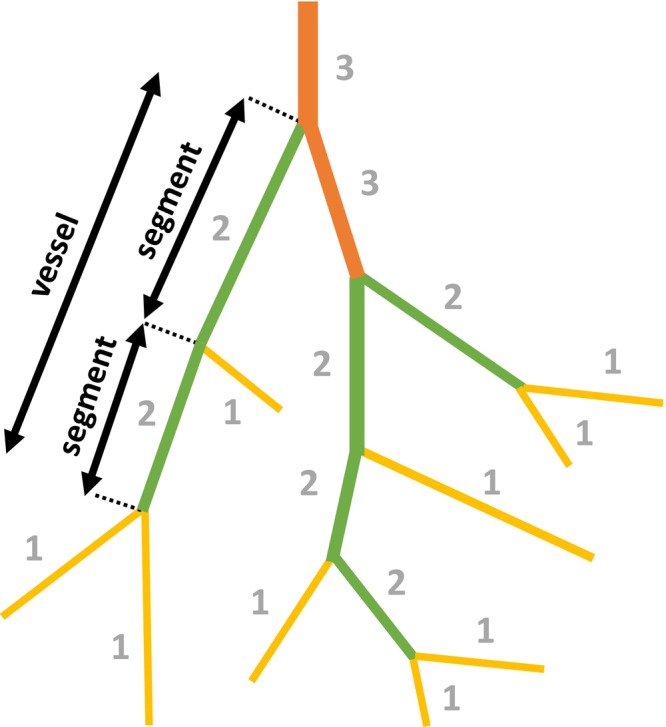
An illustration of a network colored and labeled based on Strahler's order. Yellow, orange, and green vessel segments correspond to Strahler's orders 1, 2, and 3, respectively. In accordance with Strahler's order, all terminal vessels are labeled as order 1. Parent vessels are assigned the next higher order if the two coinciding daughters have the same order; otherwise, the parent vessel is assigned the higher of the two daughters' orders. Sequentially connected vessel segments of the same order are defined as a vessel element.

Analysis of geometric and hemodynamic properties across vessel generations in the resulting networks was facilitated by grouping vessels by Strahler's and centrifugal order (Ellsworth et al., 1987; Koller et al., 1987). Both ordering schemes were used to allow for more representative comparisons to different experimental datasets when validating the algorithm's accuracy, since prior work suggests that ordering schemes are not interchangeable and will result in differing descriptions of the network (Bao et al., 2024). Additionally, these order schemes were necessary for defining vessels in this study. The algorithm approximates vessels as a sequence of straight cylindrical tubes connected in series and of the same order (Bao et al., 2024; Lapi et al., 2019; VanBavel & Spaan, 1992). Defining vessels as such allows for consideration of vessel curvature when calculating network geometry and hemodynamics statistics. For clarity, Figure 2 demonstrates the concept of segments and vessels as defined by Strahler's order in a small, illustrated network.

Network geometry was analyzed based on the diameter, length, and number of vessels per order. Additionally, the fractal dimension of the generated networks was found using the box counting method (Glenny et al., 1991; Mancardi et al., 2008; Smith et al., 1996) as a metric for network space‐filling and self‐similarity. A custom MATLAB code was developed to generate cuboid boxes of varying sizes to cover the volume of the network without overlap. For each linearly increasing scale *s*, a scale factor $\mathrm{sf}=2^{s}$ was defined such that cuboid box dimensions were scaled down logarithmically ($L=\frac{L_{0}}{\mathrm{sf}}$, where L is the updated edge length of the cuboid box, and L_0_ is the minimum edge length required for a single cuboid box to encapsulate the entire network, that is, the minimum bounding cuboid box). The number of cuboid boxes were increased at each scale to cover the entire network within the volume of the minimum bounding cuboid box. Fractal dimension (D_F_) could then be calculated as the slope of the linear fit between the logarithm of the number of boxes containing the network (N) and the logarithm of the scale factor, which is representative of the cuboid box size.

$$D_{F}=\frac{\log\left(N\right)}{\log\left(\mathrm{sf}\right)}$$

Network hemodynamics were analyzed based on blood flow, discharge, and tube hematocrit over vessel diameter. Perfusion heterogeneity (γ) was calculated as the fractional flow down each daughter arteriole arising from their parent (i.e., γ of 0.5 reflects homogenous flow; values deviating from 0.5 reflect increasingly heterogenous perfusion across any arteriolar bifurcation); this metric has been applied in prior work where increasingly heterogeneous distributions of blood flow at successive arteriolar bifurcations have been associated with pre‐capillary arterioles having low perfusion, low hematocrit, and diminished capacity to regulate perfusion (Frisbee et al., 2016). Additionally, the presence of certain relationships between geometric and hemodynamic properties, as previously observed in experimental studies, was investigated. These relationships include Horton's law (Woldenberg, 1986), Murray's law (Murray, 1926), and the Fahraeus and network Fahraeus effects.

In this study, Horton's law was evaluated in generated networks based on the strength of the linear fit between the log transform of mean diameter, length, and number of vessels per Strahler's order. Horton's ratios were also calculated using three different methods typically observed in literature: Method 1: the ratios are calculated as the slope of the linear fit on the logarithmic transform of the mean diameter, length, and number of vessels per Strahler's order of all generated networks. Method 2: the ratios are calculated as the average slope of the linear fit on the logarithmic transform of the mean diameter, length, and number of vessels per Strahler's order of each individual network. Method 3: the mean diameter, length, and number of vessels per Strahler's order of each individual network are averaged between all generated networks. The ratios are then calculated as the slope of the linear fit on the logarithmic transform of these averaged means.

Murray's law was evaluated based on the Murray's law exponent, calculated as the slope of the linear fit to the logarithmic transform of vessel segments' diameter and blood flow from the networks of interest. Vessel segments were individually evaluated in this case instead of full vessels due to previous work (Bao et al., 2024), which demonstrated that the definition of vessels may vary significantly due to differences between the Strahler's and centrifugal ordering schemes. This method for finding the Murray's law exponent follows methods previously used in experimental work (Al‐Khazraji et al., 2012).

Finally, the Fahraeus and network Fahraeus effects were evaluated by observing vessel segment tube and discharge hematocrit, respectively, relative to their diameter over multiple networks, given a fixed inlet discharge hematocrit of 0.4.

### 2.3 Properties of the generated networks

**FIGURE 3:**
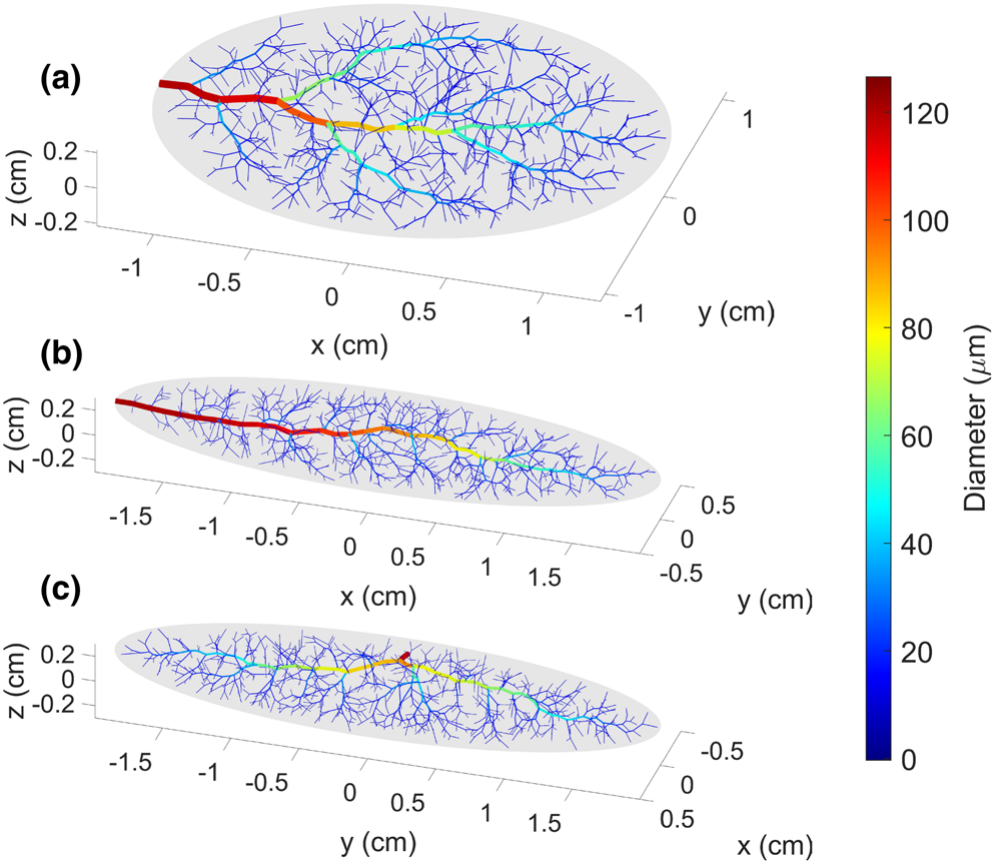
Microvascular networks generated up to 700 bifurcations within three unique tissue shapes, with vessels colored by diameter (μm). Tissue A is a wide and flat tissue (*Rx*, *Ry*, *Rz* = 6, 5, 1). Tissue B is a narrow tissue (*Rx*, *Ry*, *Rz* = 20, 5, 3). Tissue C is a narrow tissue with the same dimensions as Tissue B; however, the network enters the tissue from its midsection instead of its tip.

**FIGURE 4:**
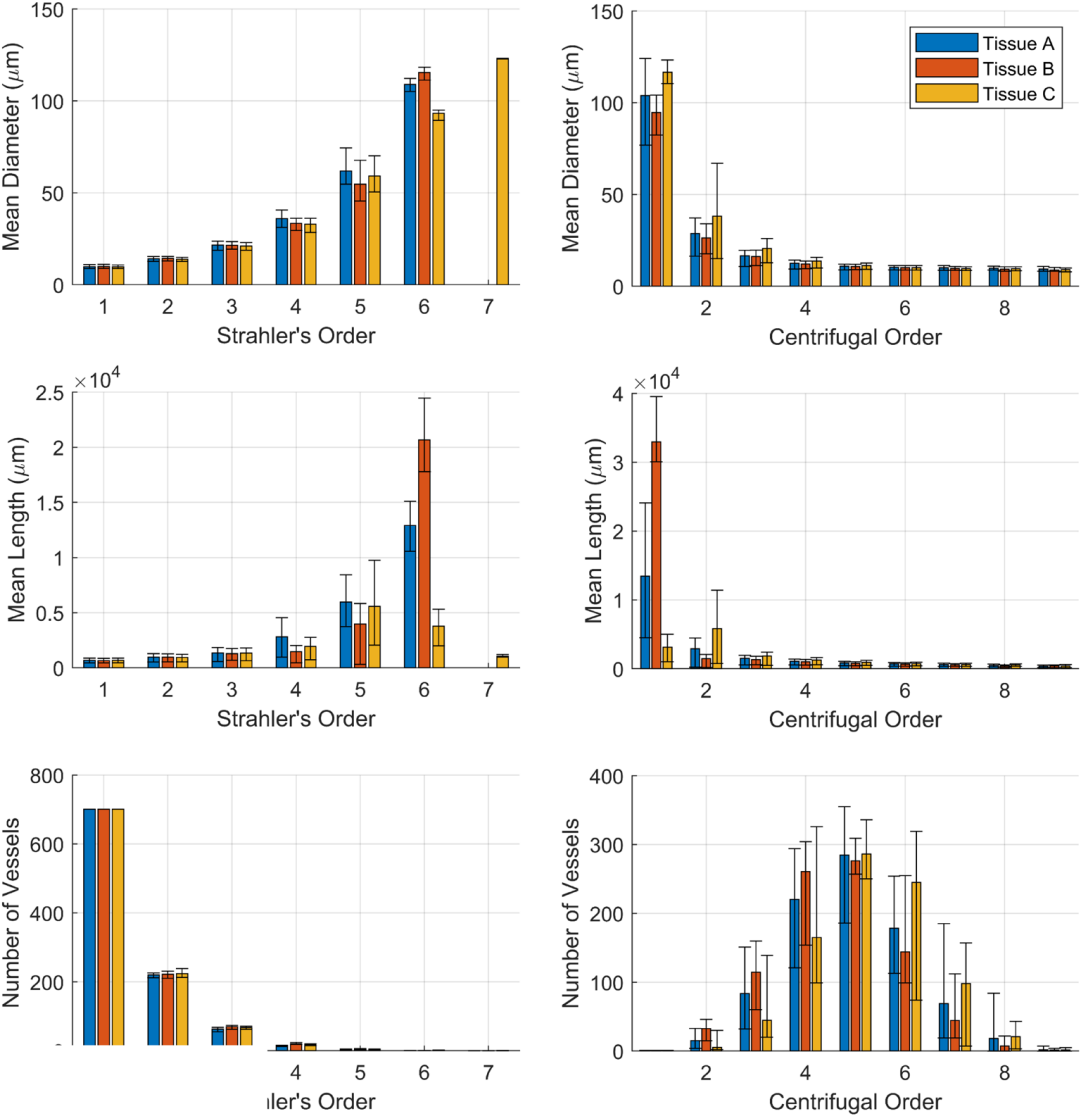
Mean diameter, length, and number of vessels per Strahler's and centrifugal order from ten 700‐bifurcation networks generated in each tissue shape A, B, and C. Error bars represent standard deviation.

**FIGURE 5:**
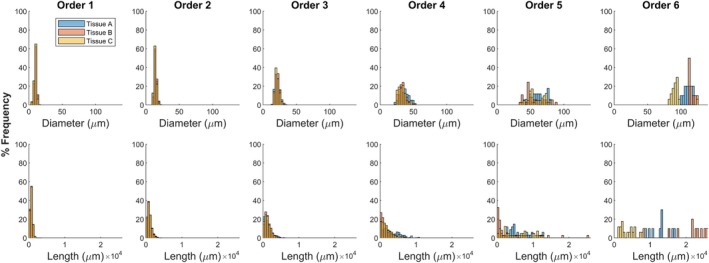
Frequency distributions of diameter and length in Strahler's order from ten 700‐bifurcation networks generated in each tissue shape A, B, and C.

**FIGURE 6:**
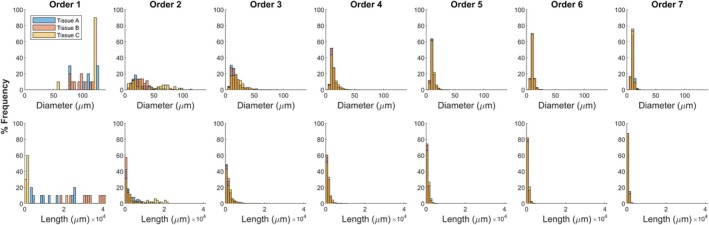
Frequency distributions of diameter and length in centrifugal order from ten 700‐bifurcation networks generated in each tissue shape A, B, and C.

**FIGURE 7:**
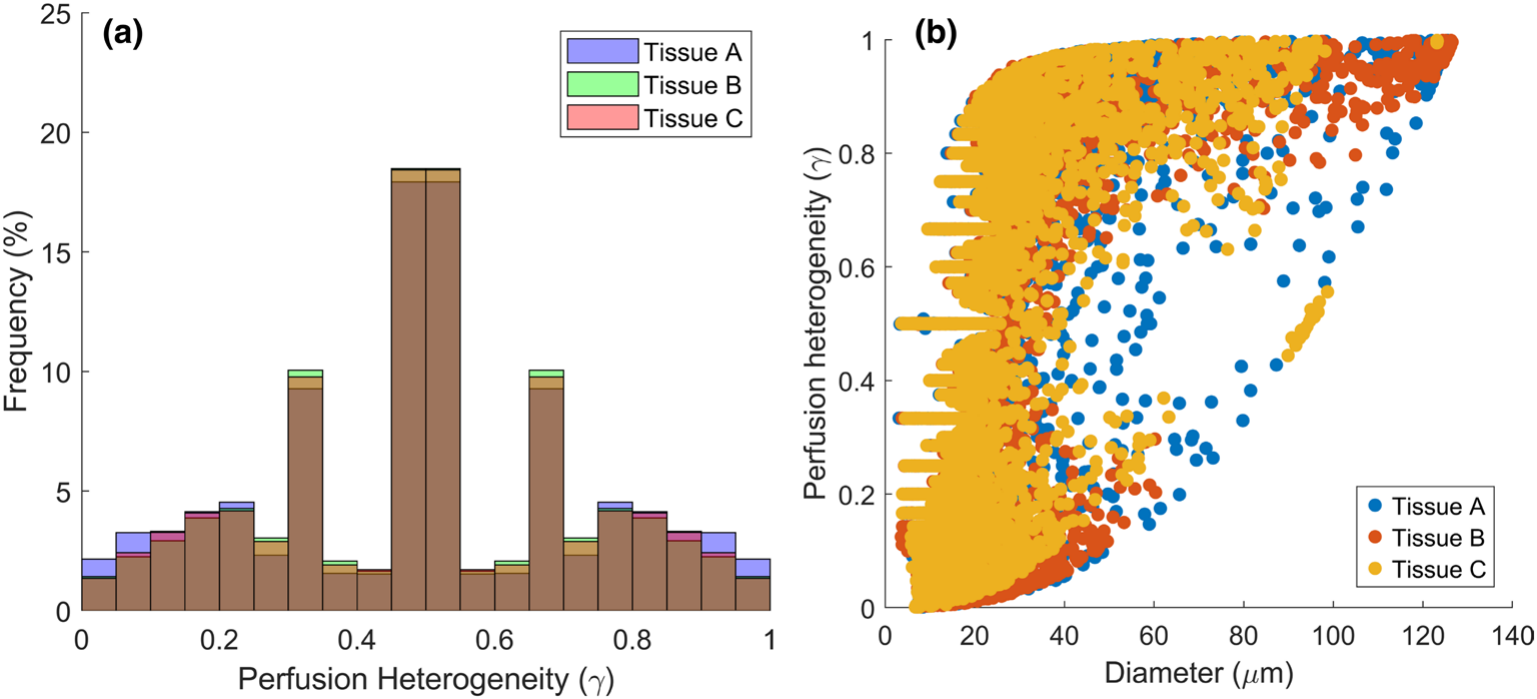
(a) Frequency distribution of perfusion heterogeneity and (b) perfusion heterogeneity plotted versus diameter for all vessels in ten 700‐bifurcation networks generated in each tissue shape A, B, and C.

**FIGURE 8:**
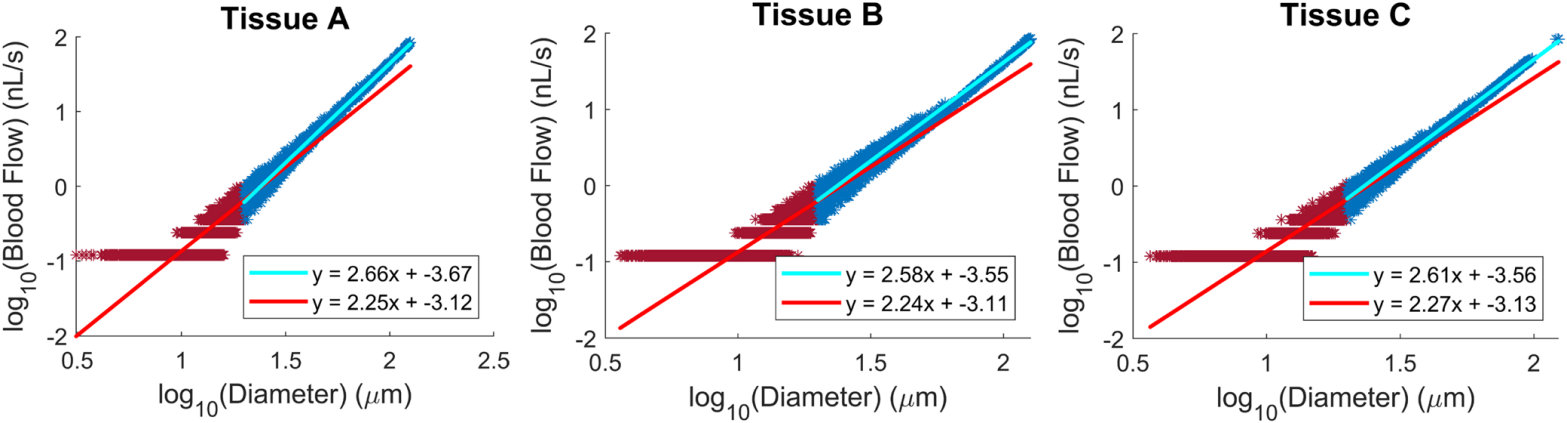
The logarithm of blood flow plotted versus the logarithm of diameter for Tissues A, B, and C. The Murray's Law exponent may be calculated as the slope of the linear fit on all vessels (red) or when excluding vessels below 20 μm (blue), to represent experimental conditions when measuring the Murray's Law exponent in vivo.

To explore the properties of networks generated in different tissue shapes, three vascularized tissues were defined based on the properties listed in Table 1, varying only in their width‐length‐height dimensions (*Rx*, *Ry*, and *Rz*). Tissue A is a wide, long, and flat tissue, in approximation of the gluteus maximus. Tissue B is a long, narrow tissue, in approximation of the spinotrapezius. Tissue C has the same dimensions as Tissue B, but the inlet vessel enters from the side of the muscle rather than the tip. These tissue shapes along with their vascular networks are depicted in Figure 3. The geometric (diameters, lengths, and number of vessels per Strahler's and centrifugal order) and hemodynamic (Murray's law exponent and hematocrit) properties of these vascular networks are presented in Figures 4, 5, 6, 7, 8.

### 2.4 Comparisons of microvascular regions

**FIGURE 9:**
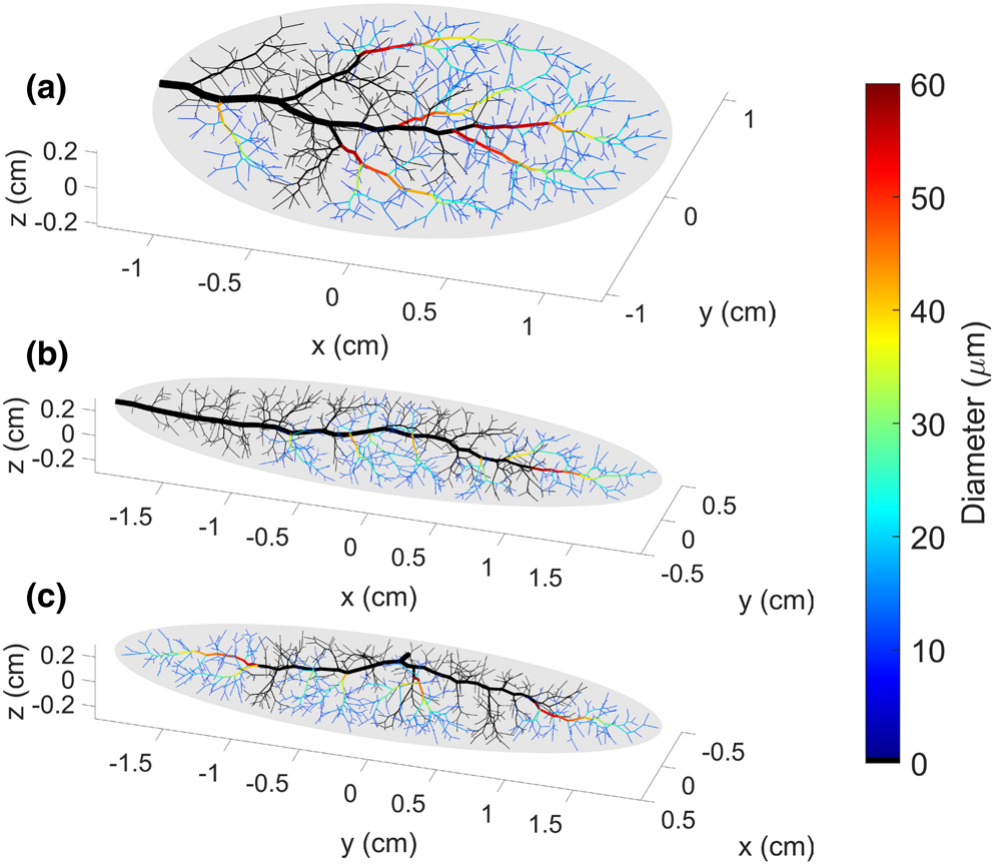
Tissues A, B, and C with only sampled subtrees colored based on diameter, while the rest of the vascular network is shown in black. It may be observed that subtrees are sampled from throughout the tissue, wherever a subtree that satisfies the condition of having an inlet vessel diameter of 50 ± 10 μm exists.

**FIGURE 10:**
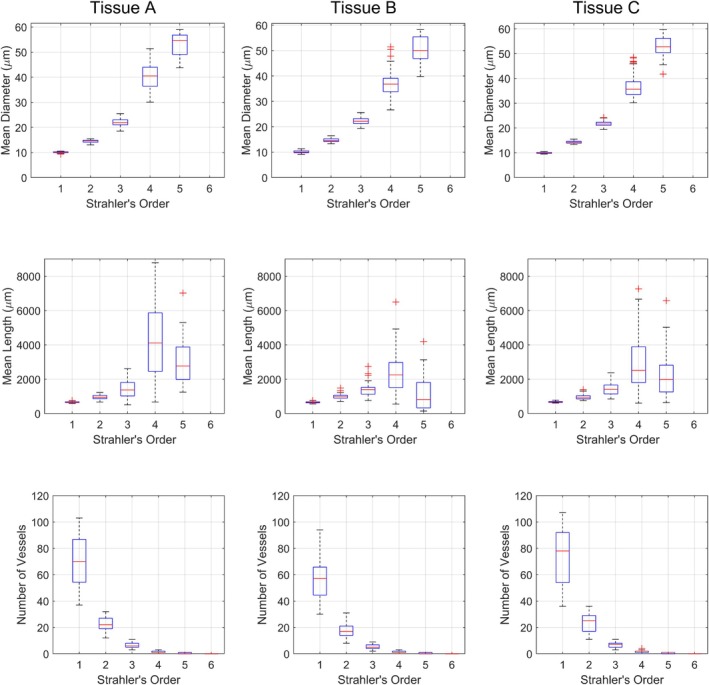
The distribution of mean diameter, mean length, and number of vessels per Strahler's order (represented as box plots) of subtrees sampled from ten 700‐bifurcation networks generated in each of Tissue A, B, and C. Each box shows the median (the central line) and the interquartile range (top and bottom of the box). Whiskers reach the furthest points that are not outliers (considered as more than 1.5 times the interquartile range away from the bottom or top of the box). Outliers are plotted individually as a “+” symbol. Variation due to the subtrees being sampled from different areas of the same tissue shape can be considered from the box width. Variation due to differences in tissue shape may be observed by comparing between figure columns.

**FIGURE 11:**
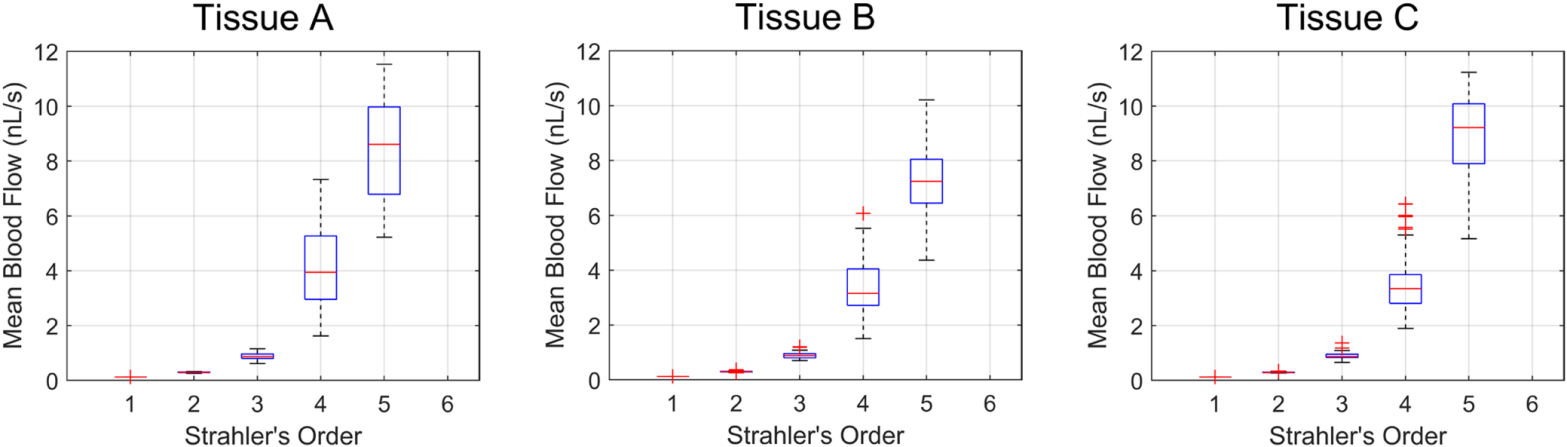
The distribution of mean blood flow per Strahler's order (represented as box plots) of subtrees sampled from ten 700‐bifurcation networks generated in each of Tissue A, B, and C. Each box shows the median (the central line) and the interquartile range (top and bottom of the box). Whiskers reach the furthest points that are not outliers (considered as more than 1.5 times the interquartile range away from the bottom or top of the box). Outliers are plotted individually as a “+” symbol. Variation due to the subtrees being sampled from different areas of the same tissue shape can be considered from the box width. Variation due to differences in tissue shape may be observed by comparing between figure columns.

To investigate whether tissue geometry affects its microvascular properties, small microvascular trees (henceforth also referred to as “subtrees”) were sampled at multiple locations across the vascularized tissue. This method reflects in vivo procedures in which small microvascular networks are selected for measurement within a larger tissue, allowing for representative comparisons with experimental data. This method also addresses issues with scale: it is uncertain whether tissue geometry will affect microvascular properties in cases where tissue bounds are magnitudes greater than its microvascular regions. Given the conventional threshold of defining microcirculation as vessels under 100 μm in diameter (Munoz et al., 2020; Ocak et al., 2016), subtrees were conservatively defined as networks ranging from 50 μm in diameter and below. An example of this process is illustrated in Figure 9 for varying tissue shapes. Comparison of subtree geometric and hemodynamic properties was made between subtrees of the same tissue, to evaluate how microvascular properties differ at different tissue locations, and between subtrees from different tissues, to evaluate whether tissue shape influences microvascular properties (Figures 10 and 11).

### 2.5 The influence of tissue size

**FIGURE 12:**
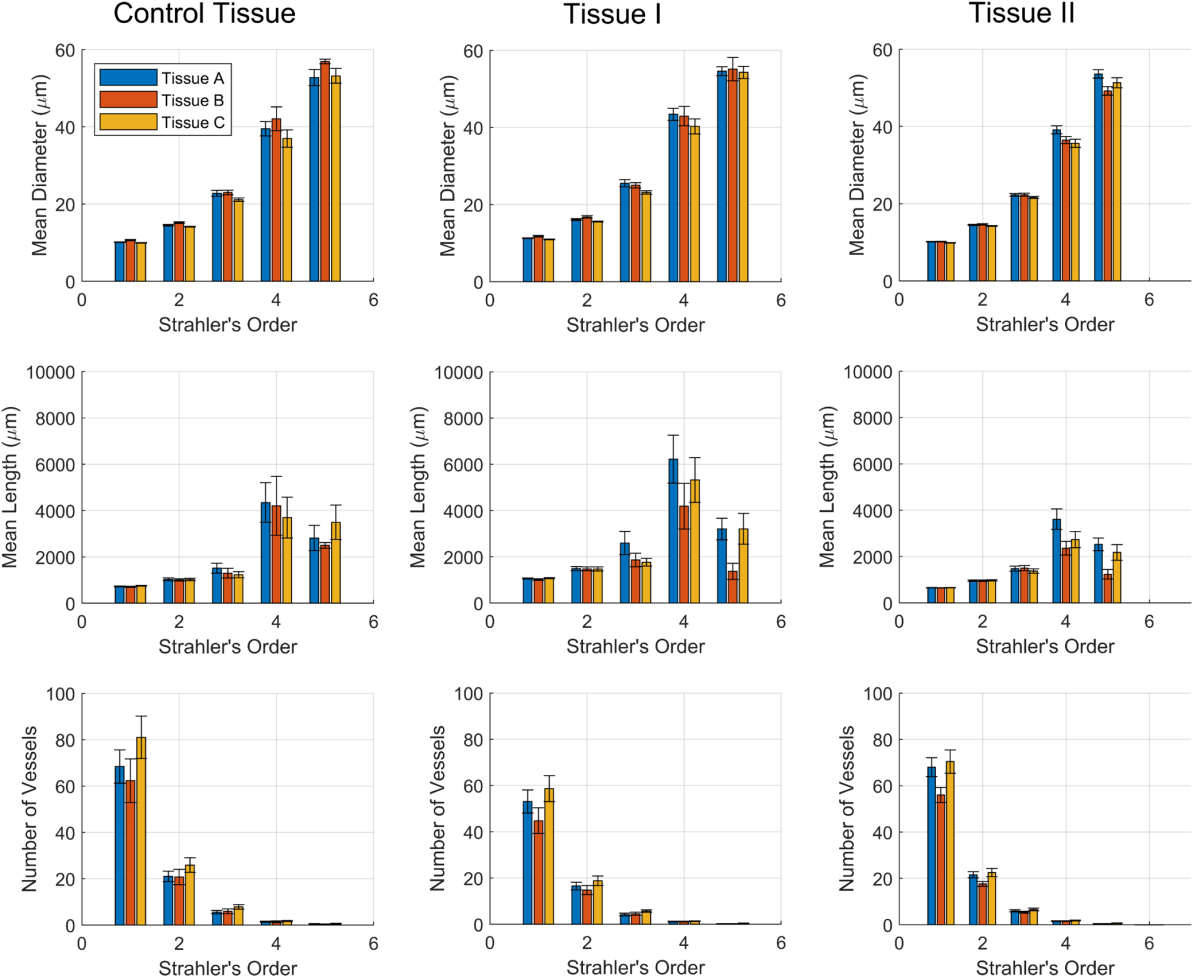
The average of mean diameter, mean length, and number of vessels per Strahler's order for subtrees sampled from each tissue shape A (*n* = 30, 36, 82), B (*n* = 16, 22, 95), and C (*n* = 29, 36, 72) for the Control Tissue, Tissue I, and Tissue II, respectively. Error bars represent the 95% confidence interval.

**FIGURE 13:**
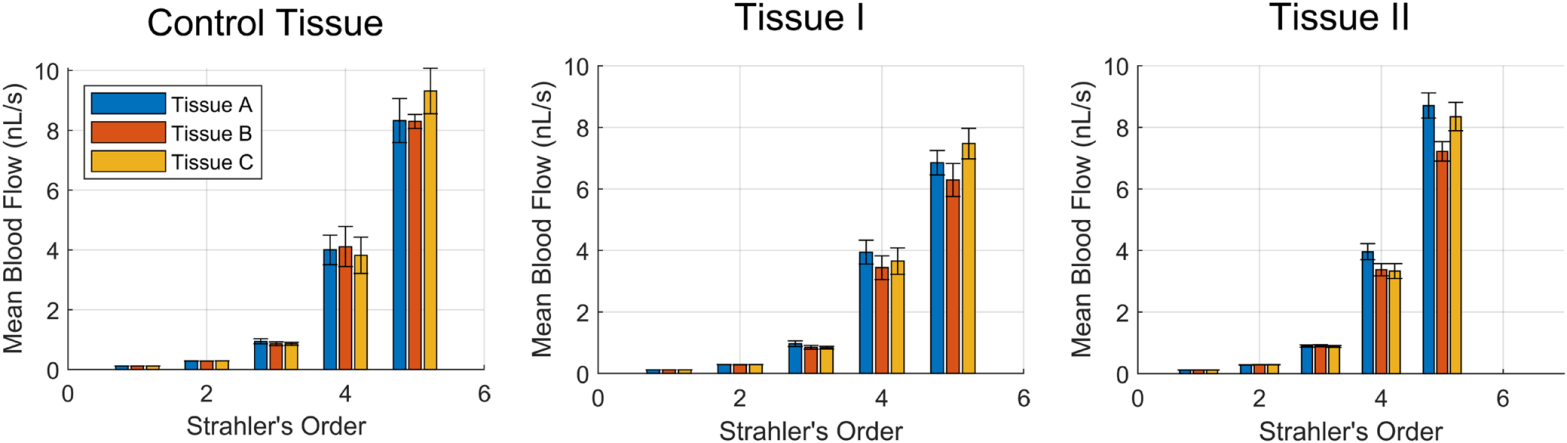
The average of mean blood flow per Strahler's order for subtrees sampled from each tissue shape A (*n* = 30, 36, 82), B (*n* = 16, 22, 95), and C (*n* = 29, 36, 72) for the Control Tissue, Tissue I, and Tissue II, respectively. Error bars represent the 95% confidence interval.

Once the influence of tissue shape on its microvascular properties was demonstrated, further investigation was conducted on the influence of tissue size. Tissue size may be a confounding factor, as the scale difference between some tissues and their microvasculature may be too significant for tissues shape to have any effect. To explore the influence of tissue size, subtrees were sampled from vasculature generated in a control tissue and two larger tissues. The control tissue was defined using parameters listed in Table 1, as they are appropriate for approximating skeletal muscle tissues. The larger tissues were defined as such: Tissue I, the tissue volume is enlarged while the vascular topology is maintained (increasing *Vperf*), and Tissue II, the tissue is enlarged while microvascular density is maintained (increasing *Nbif*). Both definitions serve different purposes: Tissue I isolates the effects of tissue size by increasing tissue volume irrespective of other variables. Though there are merits to this definition, such as for understanding of tissue size as a function and for algorithm exploration, the process can be compared to placing vasculature from the control tissue into a larger tissue volume which will inevitably introduce other vascular effects (e.g., decrease in microvascular density). Tissue II is beneficial for evaluating the effects of tissue size while holding other vascular effects constant. For comparison purposes, Tissue I and II were defined to have the same total tissue volume, as summarized in Table 4. Subtrees sampled from the control tissue, Tissue I and II were analyzed for all tissue shapes A, B, and C to comprehensively evaluate the relation between tissue shape and size and its microvascular properties (Figures 12 and 13). Total tissue volume for both definitions of the large tissue was maintained at three times the control tissue (0.54 × 3 = 1.61 cm^3^) for comparison purposes.

**FIGURE 14:**
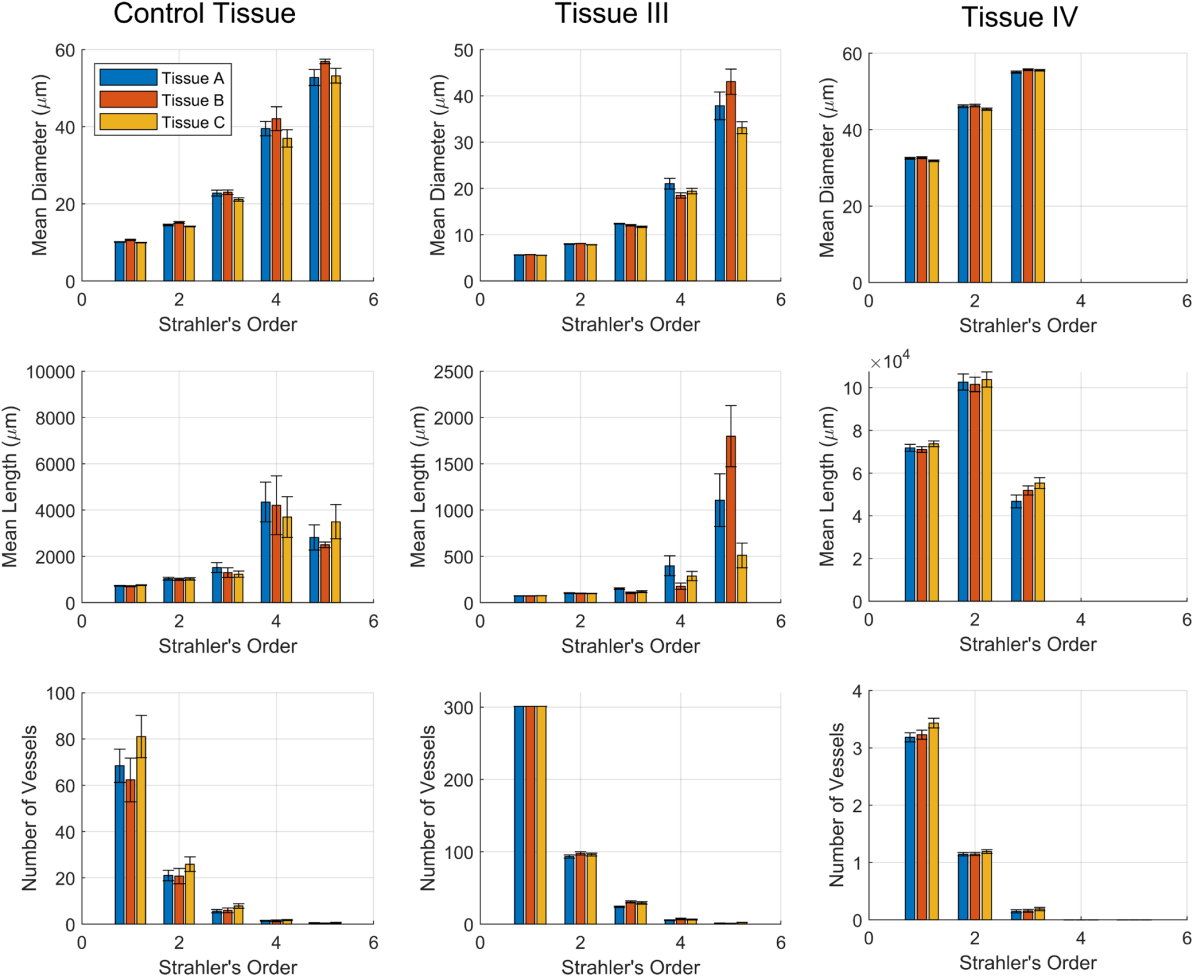
The average of mean diameter, mean length, and number of vessels per Strahler's order for subtrees sampled from each tissue shape A (*n* = 30, 10, 694), B (*n* = 16, 10, 789), and C (*n* = 29, 10, 722) for the Control Tissue, Tissue III, and Tissue IV, respectively. Error bars represent the 95% confidence interval.

**FIGURE 15:**
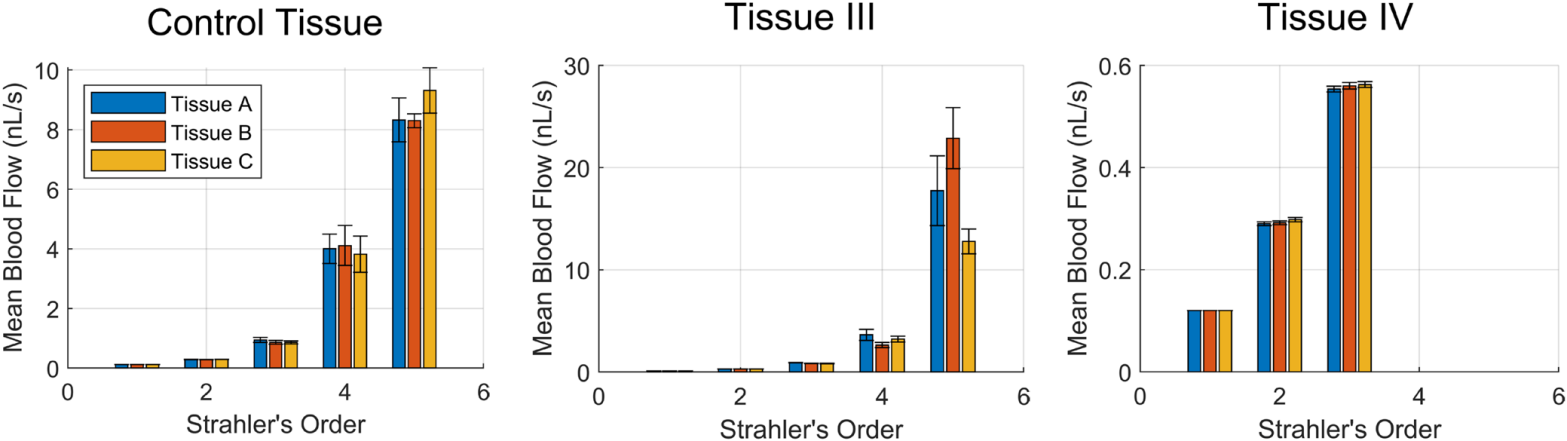
The average of mean blood flow per Strahler's order for subtrees sampled from each tissue shape A (*n* = 30, 10, 694), B (*n* = 16, 10, 789), and C (*n* = 29, 10, 722) for the Control Tissue, Tissue III, and Tissue IV, respectively. Error bars represent the 95% confidence interval.

The tissues explored in Table 4 isolate the effect of tissue shape and size on microvasculature and present a range of tissue volumes common in rodents. However, the presented range may not encompass all tissue volumes typically seen in experimental work and animal studies (e.g., skeletal muscles in humans are commonly 100–300 cm^3^ (Belavý et al., 2011)). There are also more extreme (e.g., the blue whale has tissue volumes up to 70 m^3^) or study‐specific cases (e.g., microtumors can be as small as 1 mm^3^) worth considering. As such, an additional pair of tissues, Tissue III and Tissue IV, were defined to encompass a wider range of tissue volumes. Like Tissue II, Tissue III and IV were defined by adjusting tissue volume irrespective of other variables, allowing for investigation of tissue size as a function over a more extensive range compared to the tissues of Table 4. It should be noted that, since Tissue III and IV are defined similarly to Tissue II, other vascular effects may be observed. While defining larger tissues using *Nbif* (as with Tissue I) was considered to hold microvascular density constant, the desired tissue volume range could not be tested due to challenges with computation time and algorithm stability when *Nbif* > 900. Tissue III and IV represent the largest and smallest tissues that can be reliably generated by the algorithm, with a total tissue volume of around 0.54 mm^3^ and 0.54 m^3^, respectively, placing them outside of the volume range of most studied tissues. The properties of Tissue III and IV are summarized in comparison to the control tissue in Table 5. To evaluate changes in microvascular properties over a greater range of tissue volumes, subtrees sampled from the control tissue, Tissue III and IV were analyzed over a range of geometric and hemodynamic properties and compared for all tissue shapes A, B, and C (Figures 14 and 15).

### 2.6 Algorithm validation

The algorithm was validated by comparing generated and experimental microvascular data in two rat skeletal muscles: the gluteus maximus (Al Tarhuni et al., 2016; Al‐Khazraji et al., 2012) and spinotrapezius (Engelson et al., 1985). These datasets were chosen for their high level of quantitative detail and distinct tissue shapes, allowing for assessment of the algorithm's accommodation of different tissue geometries. Validation was achieved if the generated networks reproduced experimentally observed laws (Murray's, Horton's, Fahraeus, and network Fahraeus) and matched measured geometric and hemodynamic properties.

**FIGURE 16:**
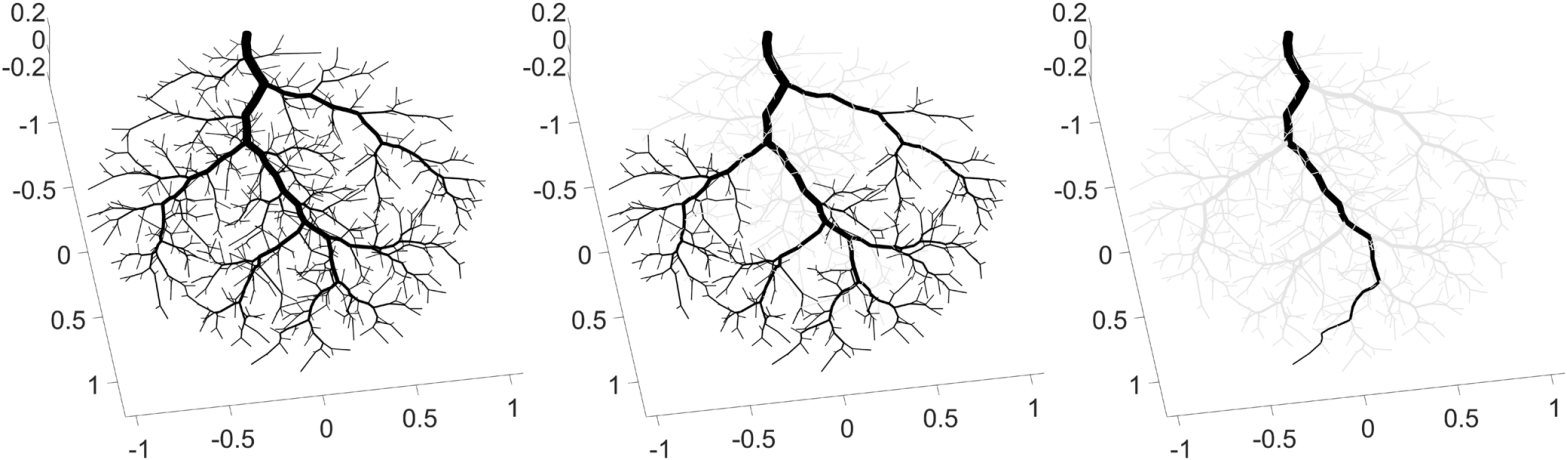
Demonstration of the user‐adjustable parameter *expVal*. From left to right, the same network is depicted with *expVal* set to 0%, 40%, and 90%. It may be observed that a higher *expVal* will result in more excluded vessels (colored gray, instead of black). Axes are labeled in arbitrary units.

Networks were generated for each muscle using different methodologies to accommodate experimental conditions. Measurements for the gluteus maximus dataset originate from intravital video microscopy (IVVM) capturing microvasculature in a small tissue area (Al Tarhuni et al., 2016). As such, for the gluteus maximus, user‐adjustable input parameters were set such that the algorithm replicates the small volume of tissue captured by IVVM. Rationale for user‐adjustable parameters was as follows: blood flow within each terminal arteriole (*Qperf*) was set to 0.12 nL/s using the diameter‐flow relation derived by Al‐Khazraji et al. (2012), assuming terminal arterioles have an average diameter of 9 μm (Al Tarhuni et al., 2016). Total pressure gradient across the network (*dPtot*) was approximated to be 30 mmHg, based on measurements in other rat skeletal muscle (Popel, 1987). Tissue volume perfused per terminal arteriole (*Vperf*) was approximated to be 1.8 mm^3^ based on total tissue volume divided by the number of terminal arterioles in the tissue; total tissue volume was set to 2 cm^3^ based on IVVM images (Al Tarhuni et al., 2016), and the number of terminal arterioles was calculated by total network blood flow (derived using the diameter‐flow relation (Al‐Khazraji et al., 2012), assuming an inlet vessel diameter of 130 μm (Al Tarhuni et al., 2016)) divided by blood flow within each terminal arteriole (*Qperf*). Tissue width‐length‐height dimensions (*Rx*, *Ry*, and *Rz*) and inlet vessel location were approximated based on IVVM images (Al Tarhuni et al., 2016) and sketches of rodent (mouse) neurovascular alignment (Engelson et al., 1985). The Murray's law exponent (γ) was set to 2.63 based on flow measurements in the rat gluteus maximus (Al‐Khazraji et al., 2012). Due to the experimental difficulties of measuring microvessels (e.g., limitations in imaging resolution), oftentimes published experimental data represent an incomplete network. Vessels sprouting from extremely asymmetric bifurcations can be especially difficult to measure. For validation purposes, an additional parameter *expVal* was introduced to better simulate the experimental conditions in which the smaller daughter vessel at bifurcations may be overlooked. The parameter *expVal* represents a percentage value, where any daughter vessel with a diameter less than this percentage value of their parent's diameter is ignored in the generated network (illustrated in Figure 16). Unless otherwise specified, *expVal* was generally set to 0 such that the presented geometric and hemodynamic network properties represent a complete network without any excluded vessels. All user‐adjustable parameters are summarized in Table 1. It may be observed that most gluteus maximus values used for validation were also used for other investigations in this study (e.g., when exploring the influence of shape and size), as they are experimentally realistic values for approximating most rat skeletal muscle vasculature.

For the spinotrapezius, rather than replicating experimental conditions as with the gluteus maximus, the whole spinotrapezius muscle was vascularized. Then, subtrees from the spinotrapezius were sampled and compared to experimental data. This methodology is more comparable to experimental procedures for collecting microvascular data, whereas the previous methodology focuses on simulating an area of interest within tissue. Subtrees were sampled as ranging in vessel diameter from ~15 μm and below in accordance with experimental data (Engelson et al., 1985). User‐adjustable parameters were defined as such: Blood flow within each terminal arteriole (*Qperf*) was set to 0.026 nL/s using the diameter‐flow relation derived by Al‐Khazraji et al. (2012), assuming terminal arterioles have an average diameter of 5 μm (Engelson et al., 1985). Total pressure gradient across the network (*dPtot*) was approximated to be 30 mmHg (Popel, 1987). Tissue volume perfused per terminal arteriole (*Vperf*) was approximated to be 0.0036 mm^3^ based on a measured average distance of 190 μm between arterioles in the spinotrapezius (Engelson et al., 1985). Tissue width‐length‐height dimensions (*Rx*, *Ry*, and *Rz*) and inlet vessel location were defined based on sketches of rodent (mouse) neurovascular alignment (Engelson et al., 1985). The Murray's law exponent (γ) was set to 2.63 (Al‐Khazraji et al., 2012). The parameter expVal was not used for the spinotrapezius (set to 0) due to differences in experimental conditions.

## 3 RESULTS

### 3.1 Properties of the generated networks

As previously mentioned, all results in this section were calculated for ten 700‐bifurcation networks generated in each of tissue shapes A, B, and C, respectively, as depicted in Figure 3. All other user‐adjustable parameters were defined based on Table 1.

The mean diameter, mean length, number of vessels per Strahler's, and centrifugal order of the 10 networks are depicted in Figure 4 for each tissue shape. It may be observed that the networks' geometric properties are comparable at most orders, with differences being most apparent at higher Strahler's/lower centrifugal orders (both of which correlate with higher vessel diameters). It may also be observed that the number of vessels per Strahler's order is highly similar between tissue shapes. This can be attributed to the Strahler's ordering scheme being reliant on network topology. Since the algorithm is designed to generate branching networks, all networks share similar topology regardless of tissue shape.

Figures 5 and 6 depict the frequency distribution of diameters and lengths of vessels per Strahler's and centrifugal order across the 10 networks generated in Tissues A, B, and C. In agreement with Figure 4, differences are more apparent in higher Strahler's orders/lower centrifugal orders. The distribution of vessels at each order resembles distributions measured in vivo for the rat gluteus maximus microvasculature (Al Tarhuni et al., 2016).

The generated vasculature in all three tissue shapes successfully demonstrates Horton's law (strong linear fit for all geometric parameters, *R* ^2^ > 0.9). Table 2 depicts the diameter, length, and bifurcation ratios for 10 networks generated in Tissues A, B, and C. The ratio values presented in the table are the average of the three methods of calculation, as differences in ratio values were insignificant (coefficient of variation <0.15). It may be observed in Table 2 that the three tissue shapes have a comparable diameter ratio. Length and bifurcation ratios show more variation however, likely due to differing tissue boundary constraints.

**TABLE 2:** The Horton's law ratios for ten 700‐bifurcation networks averaged over the three different methods, presented for each tissue shape and for both centrifugal and Strahler's orders.

Tissue shape	Ratios (Centrifugal|Strahler's)					
	Diameter (R_D_)		Length (R_L_)		Bifurcation (R_B_)	
A	1.4190	1.6247	1.5964	1.8211	1.9605	3.8081
B	1.3911	1.6163	1.6735	1.8584	1.7163	3.7591
C	1.5005	1.5873	1.4238	1.5049	2.3241	3.4721

Table 3 depicts the fractal dimension of the networks generated in Tissues A, B, and C across multiple scale factors. Bold values correspond to the strongest linear fit (*R* ^2^ > 0.95) between the logarithm of the number of boxes over the logarithm of the scale factors, suggesting the most accurate fractal dimension. It may be observed that their fractal dimensions are comparable, being around 2.1; this may be due to all networks being generated within a relatively empty 3D ellipsoidal space. Fractal dimension decreases from Tissue A to B to C. With all other parameters being equal, these differences in fractal dimension likely originate from differences in tissue shape and inlet vessel position (Tissue B vs. C). As previously mentioned, fractal dimension is associated with how an object fills in space; fractal dimension is closer to 3 if the object occupies a 3D space, closer to 2 if the object occupies a surface, and closer to 1 if the object occupies a line. The fractal dimension of the networks in Tissue A being greater than Tissue B and C suggests its vasculature occupies a 3D space more completely. This is reasonable as Tissues B and C are both shaped narrowly and more akin to a line geometrically. Meanwhile, Tissue B having a greater fractal dimension than C suggests that changes in inlet vessel positioning affect the network's ability to fill in the space; the inlet vessel entering through the long side of the tissue and splitting into two daughter vessels upon contact with tissue boundaries appears to fill in space less efficiently compared to the inlet vessel entering through the tip of the tissue.

**TABLE 3:** Average fractal dimension of ten 700‐bifurcation networks generated in each Tissue A, B, and C, and calculated over various scale factors.

Scale factor	Tissue A	Tissue B	Tissue C
1	0	0	0
2	1.8372	1.0322	1.3949
3	2.1293	1.8772	1.9185
4	**2.1981**	2.0909	2.0779
5	2.0478	**2.1363**	**2.1106**
6	1.7689	1.9771	1.9516

*Note*: Bold is the fractal dimension most representative of the respective tissue, based on strength of the linear fit to the logarithm of the number of boxes over the logarithm of the scale factor (*R* ^2^ > 0.95).

**FIGURE 17:**
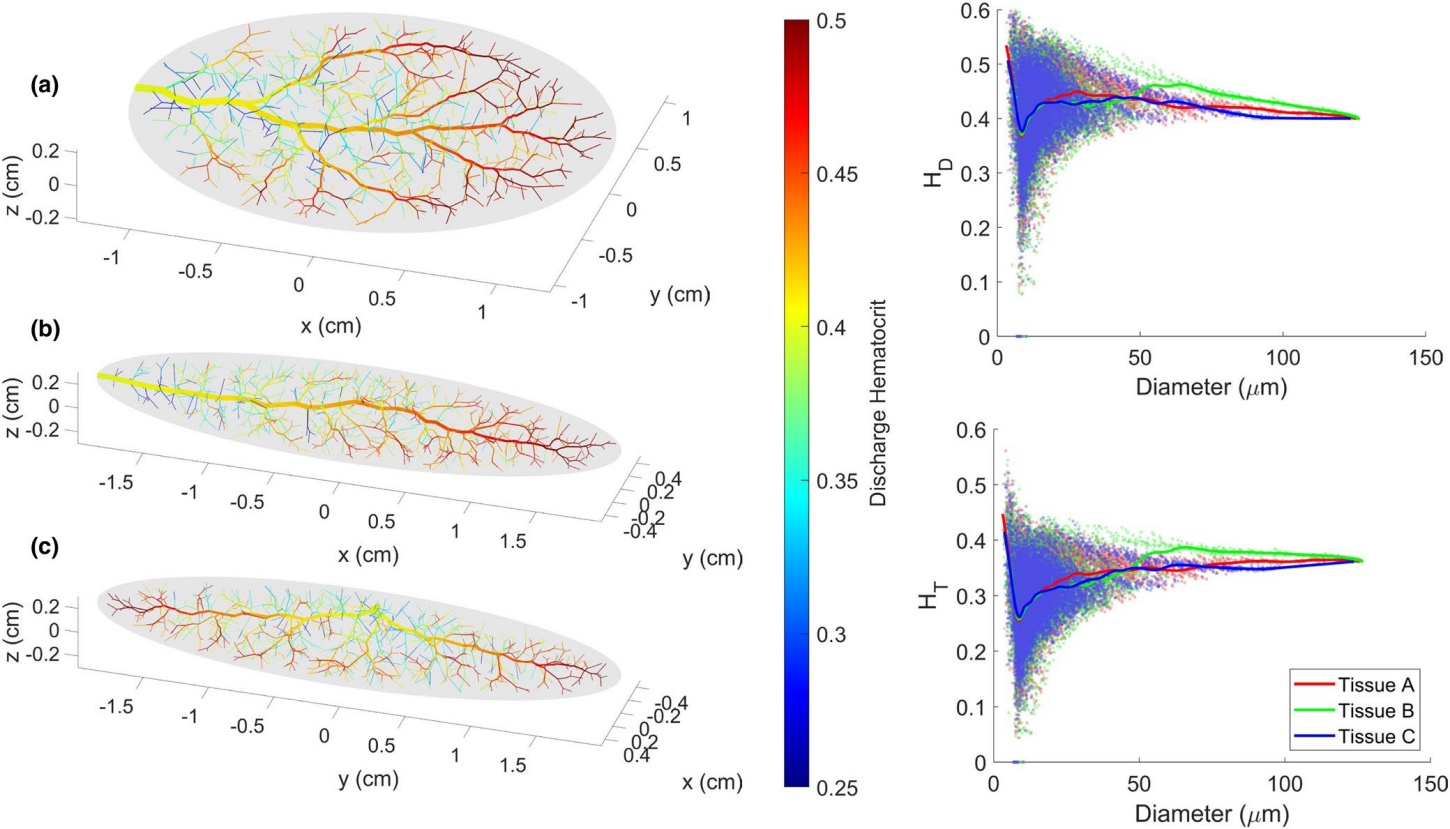
On the left, 700‐bifurcation vascular networks in Tissues A, B, and C are shown with vessels colored by discharge hematocrit. On the right, vessel discharge and tube hematocrit are plotted versus diameter accumulated from ten 700‐bifurcation networks generated in each of Tissue A, B, and C. A cubic spline has been applied to show the general trend.

Hemodynamic properties are highly similar between tissues A, B, and C despite differences in shape. Perfusion distribution is highly homogenous across all three tissue shapes (Figure 7a), with gamma values being centered and symmetrically distributed around 0.5. In Figure 7b, a notable trend can be observed for all three tissue shapes, wherein perfusion is increasingly heterogeneous as vessel diameters decrease. Other similarities between the three tissue shapes include their Murray's law exponent (Figure 8), as well as their discharge and tube hematocrit values, which follow the Fahraeus and network Fahraeus effect as observed in vivo (Figure 17). It may be noticed in Figure 17 that discharge hematocrit distributions vary depending on network shape: Tissue B has higher discharge hematocrit (H_D_) at its tip opposite to the inlet vessel, whereas Tissue C accumulates higher H_D_ at both ends of the tissue. Meanwhile, Tissue A has higher H_D_ around the edges of the tissue. Though this distribution suggests that the shape of the tissue and its accompanying vasculature influences microvascular hemodynamics, it is uncertain whether this H_D_ gradient (which generally ranges from ~0.37 to 0.43) is significant enough to have physiological effects.

### 3.2 Comparisons of microvascular regions

Figure 9 presents an example of how subtrees were sampled from networks generated in each tissue shape A, B, and C. These subtrees are colored by vessel diameter; all other vessels are black. For ten 700‐bifurcation networks generated in each tissue shape A, B, and C respectively (all other user‐adjustable parameters defined based on Table 1), 25 subtrees were sampled from A, 12 from B, and 22 from C.

Figure 10 presents geometric properties of all sampled subtrees as box plots. The box plots themselves represent the variation between subtrees sampled from different areas of tissues, whereas variations due to tissue shape can be observed between the figure's columns. It can be observed that subtrees sampled from different locations in the tissue vary at higher Strahler's orders but become comparable around Strahler's order 3 and below (which corresponds to vessel diameter ~25 μm and below). Similarly, it may be observed by comparing between figure columns (corresponding to tissue shapes A, B, and C) that differences in microvascular properties become insignificant at Strahler's order 3 and below.

To understand the hemodynamics of subtrees sampled from Tissues A, B, and C, Figure 11 presents the distribution of blood flow across Strahler's orders of the sampled subtrees. Again, it may be observed that differences at Strahler's order 3 and below are insignificant. Discharge and tube hematocrit per Strahler's order was highly similar across all Strahler's orders (coefficient of variation <0.05), regardless of the tissue location at which the subtree was sampled or the tissue geometry. For brevity, H_T_ and H_D_ are not depicted in Figure 10. It may be noted that, depending on where the subtree is sampled, a small hematocrit gradient is sometimes observable (Figure 17); as mentioned previously, it is uncertain whether the gradient is significant enough to induce physiological effects.

All geometric and hemodynamic analysis and comparisons were repeated for centrifugal order, as past work has suggested that differing vessel ordering results in divergent descriptions of networks (Bao et al., 2024). For brevity, these results are not shown, as centrifugal ordering yielded similar conclusions—that variations in microvascular properties are predominantly observed in orders corresponding to vessel diameters above ~25 μm, and that microvascular properties at and below this diameter threshold were similar between different locations in a tissue as well as between different tissue shapes.

### 3.3 The influence of tissue size

Figures 12 and 13 present geometric and hemodynamic properties of networks (the average of mean diameter, mean length, and number of vessels ± the 95% confidence interval) generated within all three tissue shapes A, B, and C, based on the set of user‐adjustable parameters Tissue I and II (summarized in Table 4). Consistent with previous findings (Figures 10 and 11), differences in microvascular properties attributable to tissue shape are primarily apparent above ~25 μm in vessel diameter whereas microvascular properties below ~25 μm are comparable. Figures 14 and 15 similarly present geometric and hemodynamic properties of networks generated in all three tissue shapes A, B, and C, but over a wider range of tissue volumes as specified by Tissue III and IV in Table 5. Unlike Tissues II and III, networks generated within Tissues III and IV suggest different vessel diameter thresholds where tissue shape begins to exert influence. Differences between tissue shapes may be observed above vessel diameters of ~10 μm (Strahler's order 3) for Tissue III. No significant differences between tissue shapes may be observed for Tissue IV, suggesting that the vessel diameter threshold is greater than ~50 μm (the diameter where subtrees were defined). This also aligns with prior hypotheses that tissue shape will not affect microvascular properties in cases where the tissue's bounds are magnitudes greater than its microvascular regions.

**TABLE 4:** Network properties for the control tissue, Tissue I, and Tissue II to investigate the influence of tissue size under experimentally realistic conditions.

Network properties		Control tissue	Tissue I	Tissue II
Input	*Nbif*	300	300	900
	*Qperf* (nL/s per terminal)	0.12	0.12	0.12
	*Vperf* (mm^3^ per terminal)	1.8	5.3	1.8
	*dPtot* (mmHg)	30	30	30
Output	Length × width × height dimensions (cm) for tissue shapes A, B, C (top to bottom)	0.97 × 0.81 × 0.16	1.40 × 1.17 × 0.23	1.40 × 1.17 × 0.23
		1.50 × 0.38 × 0.23	2.17 × 0.54 × 0.33	2.17 × 0.54 × 0.33
		0.38 × 1.50 × 0.23	0.54 × 2.17 × 0.33	0.54 × 2.17 × 0.33
	Total tissue volume (cm^3^)	0.54	1.61	1.61
	Average diameter range (μm)	5–95	5–100	4–135

*Note*: Input parameters that differ from the control tissue are colored red. Vessel diameter ranges are represented as an average aggregated across all three tissue shapes (*n* = 10 networks were generated for each tissue shape in Control Tissue, Tissue I and II).

**TABLE 5:** Network properties for the control tissue, tissue III, and tissue IV to investigate the influence of tissue size under mathematical extremes.

Network properties		Control tissue	Tissue III	Tissue IV
Input	*Nbif*	300	300	300
	*Qperf* (nL/s per terminal)	0.12	0.12	0.12
	*Vperf* (mm^3^ per terminal)	1.8	1.8e‐3	1.8e+6
	*dPtot* (mmHg)	30	30	30
Output	Length × width × height dimensions (cm) for tissue shapes A, B, C (top to bottom)	0.97 × 0.81 × 0.16	0.10 × 0.08 × 0.02	97.3 × 81.1 × 16.2
		1.50 × 0.38 × 0.23	0.15 × 0.04 × 0.02	151 × 37.6 × 22.6
		0.38 × 1.51 × 0.23	0.04 × 0.15 × 0.02	37.6 × 151 × 22.6
	Total tissue volume	0.54 cm^3^	0.54 mm^3^	0.54 m^3^
	Average diameter range (μm)	4–95	2–50	15–285

*Note*: Input parameters that differ from the control tissue are colored red. Vessel diameter ranges are represented as an average aggregated across all three tissue shapes (*n* = 10 networks were generated for each tissue shape in control tissue, tissue III and IV).

**FIGURE 18:**
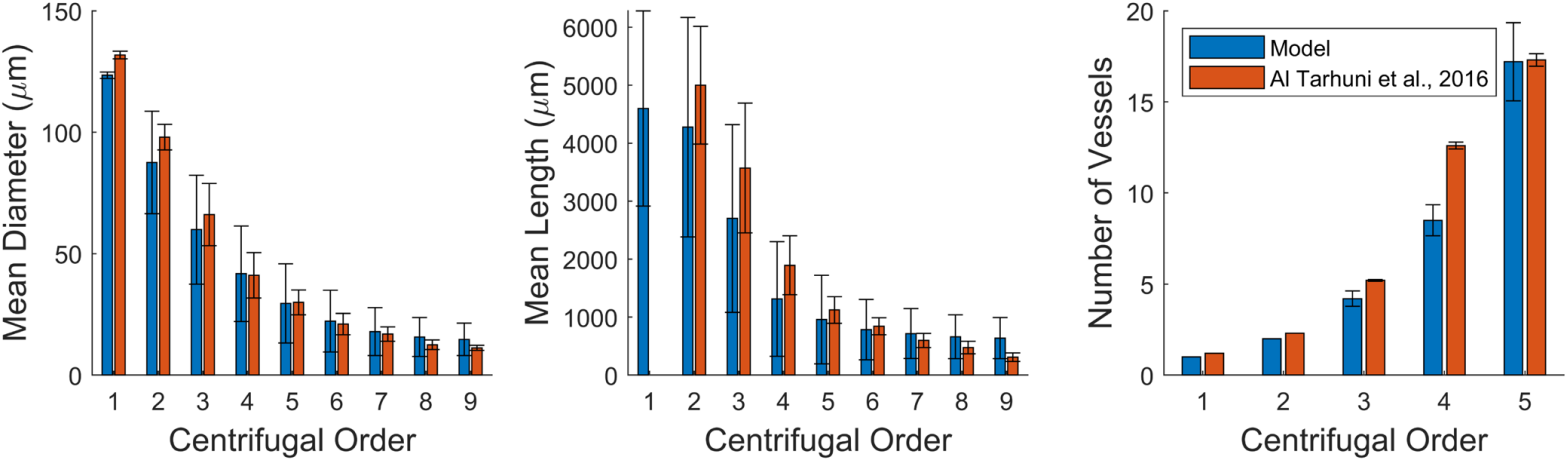
Comparison of mean diameter, length, and number of bifurcations per centrifugal order between ten 700‐bifurcation model networks in blue and experimental data (Al Tarhuni et al., 2016) in red. Error bars represent standard deviation.

**FIGURE 19:**
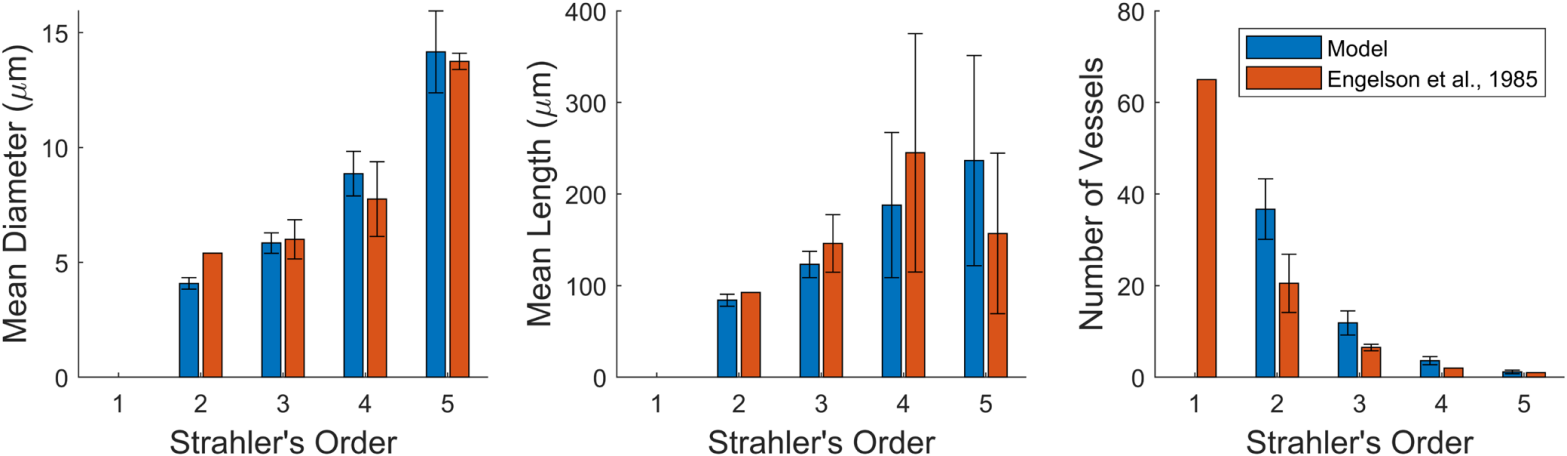
Comparison of mean diameter, length, and number of bifurcations per Strahler's order between 108 subtrees sampled from ten 700‐bifurcation model networks in blue and experimental data (Engelson et al., 1985) in red. Error bars represent standard deviation.

### 3.4 Algorithm validation

It may be observed in Figure 18 that generated networks are comparable to experimental measurements of microvasculature within rat gluteus maximus for all orders in which geometric data was available. Figure 18 supports the algorithm's ability to generate networks that are structurally accurate to rat gluteus maximus networks as observed via IVVM.

Generated networks also compared favorably with microvascular properties of the spinotrapezius dataset, as seen in Figure 19. Since experimental data for order 1 is not available for certain geometric properties, the generated network has been compared starting from Strahler's order 2. Model data in Figure 19 represents the average of 108 subtrees sampled from 10 tissues, with each subtree ranging between 14 and 20 μm in vessel diameter. Figures 18 and 19 support the algorithm's ability to generate realistic networks for different rat skeletal muscles while accommodating experimental conditions.

## 4 DISCUSSION

To summarize, this study presents a highly customizable algorithm for generating biophysically accurate vascular branching networks in different 3D tissue spaces. Using this algorithm, geometric and hemodynamic properties of networks generated within a range of tissue shapes and sizes were analyzed to understand the relationship between tissue geometry and its vascular properties. The microcirculation is of particular interest in this study, and thus to mimic experimental procedures, small arteriolar trees were sampled from different locations in skeletal muscle tissues of varying shapes and sizes. A notable observation is that tissue shape influences microvascular properties only above a certain vessel diameter threshold, which itself is dependent on tissue size. All analysis was performed using both Strahler's and centrifugal ordering schemes to accommodate divergent descriptions of networks common to the literature (Bao et al., 2024). The presented algorithm was also comprehensively validated on two different skeletal muscle datasets, confirming the algorithm's ability to generate accurate networks and its flexibility to accommodate different experimental conditions.

A review of the presented vascular properties demonstrates that generated networks agree with findings from past literature. The networks demonstrated expected mathematical relations, such as Horton's law (Table 2), Murray's law (Figure 8), the vessel and network Fahraeus effects (Figure 17). Perfusion heterogeneity as shown in Figure 7a greatly resembles previously measured γ distributions of arterioles in situ; additionally, Figure 7b is consistent with previous findings in which increasingly heterogeneous distributions of blood flow at successive arteriolar bifurcations are associated with lower hematocrit (Figure 17) at pre‐capillary arterioles. Differences in network geometry and hemodynamics attributable to tissue shape were mostly observable in larger vessel diameters (Figures 4, 5, 6 and 7b), with network properties being comparable otherwise. Though a hematocrit gradient may be observed in different tissue shapes (Figure 17), which is in agreement with past experimental findings where mean capillary hematocrit is sensitive to geometric heterogeneity of the network (Pries et al., 1995), it is uncertain if the presented gradients are significant enough to have physiological effects. However, the distribution of hematocrit values within the generated networks resembles experimentally measured hematocrit distributions from various healthy rat skeletal muscle (Pries et al., 1990; Rasmussen et al., 2018).

A key observation from the generated results is that, below a certain vessel diameter threshold, different skeletal muscle tissues appear to exhibit similar microvascular structure and hemodynamics. While overall vascular geometry may vary between differently shaped skeletal muscles (Figures 4, 5, 6), these differences become negligible at lower vessel diameters, with microvascular properties converging to previously described geometric and hemodynamic trends (Figures 10 and 11) starting from vessel diameters of approximately 25 μm. Mathematically, it may be hypothesized that this diameter threshold should continue to decrease as vessels are increasingly compressed by decreasing tissue boundaries. This hypothesis aligns with previously described concerns that microvascular properties will be unaffected by tissue geometry when tissue bounds are orders of magnitude greater than microvascular regions. Analysis of networks generated based on Tissues I–IV (Figures 12, 13, 14, 15) demonstrates that the diameter threshold of ~25 μm remains similar unless the microvessel density is very small or large, confirming that microvessel density has a confounding influence on whether tissue shape will impact microvessels properties. It may be noted that the 0.54 mm^3^ to 0.54 m^3^ tissue volume range of Table 5 was chosen based on the limits of the algorithm's current capabilities (such that it would still be able to produce a stable solution) and should safely encapsulate most tissue sizes (and microvessel densities) observed in animal studies. Though Tissues III and IV have not been confirmed to be experimentally realistic, the relative relationship between the two tissues should still allow for valid conclusions pertaining to the effect of tissue shape on vascular properties in relation to tissue size as a function. Another finding of the study worth mentioning relates to the space‐filling efficiency of certain tissue shapes and vascular structures. Fractal dimension analysis suggests (Table 3) that tissue shape and inlet vessel positions impact the degree to which a network can fill in space, and that biological networks that fill isotropic spaces are more self‐similar.

This study presents a unique investigation that uses three‐dimensional mathematical modeling of vasculature and microcirculation to explore their properties in varying tissue geometries. The algorithm has been described in a detailed step‐by‐step manner such that it may serve as a guideline for continued research in computational simulations of visually and statistically realistic arteriolar networks. Its accuracy is supported by extensive validation against two experimental datasets from the literature, including previous work that remains amongst the most comprehensive for microvascular structure and hemodynamics (Al Tarhuni et al., 2016). Analysis of networks generated using the developed algorithm finds that, though geometric and hemodynamic features of skeletal muscle microcirculation vary between different vascular regions, they are statistically similar below a certain vessel diameter threshold. The physiological or functional significance of such thresholds remains speculative. This also raises the question of whether different microvascular characteristics and resolution thresholds exist for other tissues and organs with different functionalities (e.g., heart and liver). Addressing this, however, lies beyond the scope of the current paper and may require experimental data that is not readily available in the literature. Future experimental work evaluating cross‐tissue similarities in microvascular structure and hemodynamics would contribute to this study's findings.

When discussing limitations of this study, the following points should also be considered. First, reader interpretation of hemodynamic results should account for the algorithm approximation that all terminal vessels have a constant blood flow (*Qperf*). In vivo, blood flow in terminal vessels varies based on tissue metabolic state and other regulatory factors (Al‐Khazraji et al., 2012; Pries et al., 1995). In future work, varying distributions of terminal vessel blood flow should be tested (e.g., Gaussian) to confirm study findings. Second, the algorithm does not account for structural differences which could cause variations in microvessel density in different tissue regions. As such, networks produced by the algorithm should be considered as statistically accurate to vasculature in skeletal muscle tissues on average. Third, though the algorithm has been validated for skeletal muscle, it is challenging to repeat the validation for other tissues due to limited experimental data in the literature detailing vessel properties (e.g., geometry and hemodynamics) across different levels (e.g., Strahler's/centrifugal orders) of the network. Such data is important, as microvascular differences may exist in tissues with distinct functional roles (e.g., myocardium and cerebrum). It is expected that the algorithm will generate reasonably accurate vasculature in other tissues if appropriate values for adjustable parameters can be found; this can be revisited in future work as more experimental data becomes available. Fourth, although several techniques (e.g., parallel computing) have been implemented in the algorithm to improve computation time, generating a 900‐bifurcation network in three‐dimensional space still takes approximately 30 h on a standard CPU. A key computational bottleneck lies in Step 3D, where determining optimal bifurcation angles involves evaluating total network blood volume across all possible network configurations defined by points within a discretized space. In future work, gradient descent will be explored as a promising strategy for further reduction of computation time: by guiding the search along the negative gradient of total blood volume, rather than exhaustively testing every point, convergence to the correct solution should be less computationally intensive. Methods from other algorithms may also be worth exploring for improvements in computational efficiency or in vivo accuracy, such as predefining terminal vessel end points such that an optimized network geometry and topology may be generated upon them (Runions et al., 2007) or generating realistic network structures using machine‐learning assistance (Brown et al., 2024).

The algorithm, at its current state, serves as a great starting point for further computational research in the microcirculation. It is an experimentally validated framework with broad applicability to a variety of microvascular research objectives, facilitating the investigation of hypotheses that would otherwise require animal models. This algorithm thereby also contributes towards the broader goals of the replacement, reduction, and refinement in animal research. Since the current algorithm can generate vasculature in varying ellipsoidal volumes, it may be extended to more complex tissue shapes in the future. Additionally, the algorithm is well positioned for the integration of additional functionality; though the current study presents static models of healthy microcirculation, future iterations aim to present accurate microvascular network structures with user control of vascular tone integrated, such that changing hemodynamic and geometric properties in health vs. disease conditions and drug treatments may be evaluated. This will be highly beneficial for understanding how vascular mechanisms (e.g., metabolic dilation, sympathetic activation, wall shear, etc.) integrate to produce multiscale interactions across the network that give rise to blood perfusion patterns relevant to health outcomes in major, widespread diseases.

## CODE AVAILABILITY STATEMENT

All coding work for this study, including algorithm development and network analysis, was conducted in MATLAB. The algorithm is shared under the MIT License and archived at Zenodo (DOI: 10.5281/zenodo.16747595). A user manual document with detailed usage and analysis instructions is included. Documentation in the archive is licensed under CC BY 4.0.

## ETHICS STATEMENT

This study involved the development of a computational model and biosimulation. This study did not involve human participants or animals. Therefore, ethical approval and informed consent were not required.

## Data Availability

All data used in this study for the algorithm's development and validation derive from previously published sources, as cited. Requests for third‐party datasets should be directed to the original authors or publishers of the cited work. Data collected by our group have been comprehensively analyzed in previous publications, but the full dataset is not publicly available due to ongoing analytic efforts by our group.
